# A case for preference-sensitive decision timelines to aid shared decision-making in intensive care: need and possible application

**DOI:** 10.3389/fdgth.2023.1274717

**Published:** 2023-10-10

**Authors:** Beatrix Göcking, Sophie Gloeckler, Andrea Ferrario, Giovanna Brandi, Andrea Glässel, Nikola Biller-Andorno

**Affiliations:** ^1^ Institute of Biomedical Ethics and History of Medicine, University of Zurich, Zurich, Switzerland; ^2^ Department of Management, Technology, and Economics, Swiss Federal Institute of Technology in Zurich, Zurich, Switzerland; ^3^ Mobiliar Lab for Analytics at ETH, Zurich, Switzerland; ^4^ Institute of Intensive Care Medicine, University Hospital Zurich, Zurich, Switzerland; ^5^ School of Health Sciences, Institute of Public Health, Zurich University of Applied Sciences, Winterthur, Switzerland

**Keywords:** shared decision-making, decision aids, subarachnoid hemorrhage, advance care planning, surrogate decision-makers, critical care

## Abstract

In the intensive care unit, it can be challenging to determine which interventions align with the patients' preferences since patients are often incapacitated and other sources, such as advance directives and surrogate input, are integral. Managing treatment decisions in this context requires a process of shared decision-making and a keen awareness of the preference-sensitive instances over the course of treatment. The present paper examines the need for the development of preference-sensitive decision timelines, and, taking aneurysmal subarachnoid hemorrhage as a use case, proposes a model of one such timeline to illustrate their potential form and value. First, the paper draws on an overview of relevant literature to demonstrate the need for better guidance to (a) aid clinicians in determining when to elicit patient preference, (b) support the drafting of advance directives, and (c) prepare surrogates for their role representing the will of an incapacitated patient in clinical decision-making. This first section emphasizes that highlighting when patient (or surrogate) input is necessary can contribute valuably to shared decision-making, especially in the context of intensive care, and can support advance care planning. As an illustration, the paper offers a model preference-sensitive decision timeline—whose generation was informed by existing guidelines and a series of interviews with patients, surrogates, and neuro-intensive care clinicians—for a use case of aneurysmal subarachnoid hemorrhage. In the last section, the paper offers reflections on how such timelines could be integrated into digital tools to aid shared decision-making.

## Introduction

Some clinical decision-making proceeds with little needed input from the patient, but most depends critically on the preferences of the person being treated. In the intensive care unit, it can be challenging to determine which life-deciding interventions align with patients' preferences since patients are often incapacitated and other means, such as advance directives and surrogates, which have inherent shortcomings, must be relied on for decision-making (1, 2). Managing such decision-making well requires a keen awareness of the preference-sensitive instances over the course of a patient's treatment. Highlighting *when* patient (or surrogate) input is necessary can be foundational to properly supporting efforts to promote shared decision-making (SDM). The main aim of the present paper is to examine the need for the development of preference-sensitive decision timelines, and, taking aneurysmal subarachnoid hemorrhage (aSAH) as a use case, propose a model of one such timeline to demonstrate their potential form and value.

The structure of the paper is as follows: Part one explores the challenges of SDM in the context of critical care for which such timelines might be relevant; part two lays out a model timeline with the use case of aSAH; and part three explores the potential integration of such timelines into digital tools for SDM. The final section offers some reflections and concluding remarks.

### Part one: relevant challenges in ICU SDM

SDM is a process whereby clinicians, drawing on their professional judgement and the best available scientific evidence, support patients, or those making decisions on the patient's behalf, to determine which treatments best align with the patient's values and goals of care (3–5). There are various models for decision-making (6, 7), but recent work has emphasized the value of shared decision-making. There is evidence that the loved-ones of critically-ill patients prefer for decision-making to be a collaborative process shared with clinicians, especially when it comes to decisions about withdrawing life-sustaining treatment (4, 8–12). Critical care societies and healthcare organizations internationally have strongly endorsed SDM (1, 4). For example, in a policy statement from the American College of Critical Care Medicine and American Thoracic Society, the authors write, “Clinicians should engage in a shared decision-making process to define overall goals of care (including decisions regarding limiting or withdrawing life-prolonging interventions) and when making major treatment decisions that may be affected by personal values, goals, and preferences” (1). The authors of the policy statement and others have pointed out, though, that there is confusion about what precise form SDM in the intensive care unit (ICU) should take and, importantly, *when* it should occur (1). Some have looked to address this. For example, Swiss experts (13) outline decision points at which treatment goals should be reassessed, e.g., when a patient has agreed to treatment in the ICU or when hypoxic brain damage has occurred following a complication. We believe such efforts to identify key moments for SDM are essential and could be further refined given the challenges present in the ICU.

Time plays a pivotal role in SDM in intensive care. While discussion of patients’ goals and values is important for determining which critical care interventions are suitable, the urgency of patients’ needs in the ICU makes it difficult to engage in SDM; outcomes can often be tied to the timeliness of the intervention, introducing a powerful time pressure (14, 15). Indeed, there is significant evidence to suggest that ICU clinician-family conferences about treatment planning often lack important elements of SDM (1, 4). For example, Khan and Muehlschlegel show that approximately one-third of conferences did not include discussions about the patient's previously expressed preferences or values (16). Further research suggests that clinicians and surrogates do not follow existing recommendations for incorporating patients’ values and preferences in 12%–50% of ICU-family conferences about goals of care (16, 17).

Time is a relevant factor for ICU decision-making not only in regards to urgency, but also because patients’ preferences tend to evolve (18, 19). A key characteristic of shared decision making that needs to be considered is that goal of care discussions are often iterative, changing over time (14, 20). Even when impressions of the individual's goals and values remain stable, changing prognosis and the developing nature of the situation mean that treatment should be continuously re-evaluated to ensure it best reflects the patient's preferences (21–23). For example, a recent qualitative pilot study on decision-making and patient experiences of aSAH illustrated the need for systematic reassessment of the patient's will during the acute course of treatment (24). In the momentum of responding to crisis and sustaining life, treatment provided can diverge from care the patient would have wanted (25, 26).

Further complicating efforts for SDM, advance directives often have significant limitations and surrogates often struggle with their role (27–29). In their present form, advance directives often fall short of aiding patients to accurately consider their preferred future care and patients have trouble predicting the care they might want in the future as their healthcare status changes (19, 30). Moreover, advance directives sometimes lack the kind of information that clinicians and surrogates would need to assist them in determining which treatment best aligns with the wishes of an incapacitated patient (19, 31), and surrogates often feel ill-prepared (32, 33). These shortcomings matter: a study investigating retrospective agreement to treatment found that only 19% of patients surviving neurocritical care in a state of dependency would have agreed to receive the interventions that kept them alive had they had the capacity to be involved in treatment decision-making and known the outcome of the intervention (34). The consequences are experienced not only by patients, whose treatment may not reflect their preferences, but also by clinicians and surrogates. Surrogates of critical ill patients often suffer emotional distress related to the role they are asked to play (28, 29, 35) and the burden of navigating decisions around end-of-life and life-sustaining treatment has been identified as contributing to clinician burnout (36, 37). There is a need to improve the utility of advance directives and better support both clinicians and surrogates with the weight of making critical decisions on another's behalf.

In response, there have been calls to improve the support in place for these SDM processes in the context of critical care (17), and there is recognition that digital technology may have an important role to play (38). Attention has already been given to developing tools, particularly those that incorporate digital technology, to support elements of SDM, including materials to better prepare surrogates for their role (39) and aid patients in decision making (40); improved tools for prognostication to inform clinicians who carry out such conversations (38, 41); and enhanced advance directives (30). Significant efforts have also aimed at training clinicians in how to engage in SDM (42). Less attention has been given, though, to developing materials that might help address the important shortcoming identified by the American College of Critical Care Medicine and American Thoracic Society in their policy statement: recognizing *when* shared-decision making should take place. More support is needed to increase the likelihood that SDM conversations take place at key moments so that patients’ preferences are respected, especially in the context of intensive care where there are unique challenges.

### Part two: a sample preference-sensitive timeline

The following section presents a sample preference-sensitive timeline for unexpected, severe brain injury, specifically for the case of aneurysmal subarachnoid hemorrhage (aSAH). The timeline is presented in the form of a graphical aid that highlights moments when the patient's goals and values are essential for informing care. To demonstrate the suitability of aSAH for such a timeline and how the condition speaks to SDM in the ICU, aSAH is first introduced below.

aSAH is a serious, sudden medical event, associated with significant mortality rates and high survivor morbidity (43). It affects about eight individuals out of 100,000 per year (44), half of whom are younger than 55 years old (45). Patients are often unconscious or neurologically impaired and unable to express their preferences; given their relative youth, many do not have advance directives (24). It is common for those who have been treated for aSAH to remain dependent on care from others following discharge; fewer than two thirds are found to live independently at 1-year follow-up (45). Survivors often have longstanding cognitive impairments that affect memory, language, and executive function (46). Some face challenges with basic activities of daily living such as feeding, dressing, and bathing (46). Fatigue and depression are also common (46). Survivors often contend with significantly reduced quality of life. Given the high risks and burdens, aSAH is a condition where knowledge of the person's goals and values is essential when it comes to considering which interventions to pursue. Recent guidelines for the treatment of aSAH have called for an emphasis on SDM (12).

In the case of aSAH, the person's condition can evolve rapidly and unpredictably, all-the-more so highlighting the need for regularly assessing whether care aligns with the person's preferences. There are many instances in the first two weeks following the initial bleed when quick decisions are necessary. The initial response to a ruptured aneurysm is usually to secure it through surgical clipping or endovascular coiling (47). Neurological and systemic complications can then occur, including early rebleeding, most commonly within the first 24 h (12), and potential elevated intracranial pressure (48), hydrocephalus (12), seizures (49), vasospasm or delayed cerebral ischemia (50) that tend to happen within 3–14 days of the initial bleeding event (12, 51). Furthermore, aSAH patients frequently suffer from extracerebral complications such as cardiac injury, arrhythmias, and acute respiratory distress syndrome (52). Determining how to respond to these complications requires weighing the burden of the treatment and likelihood that interventions may lead to an intolerably low quality of life.

**Figure 1:**
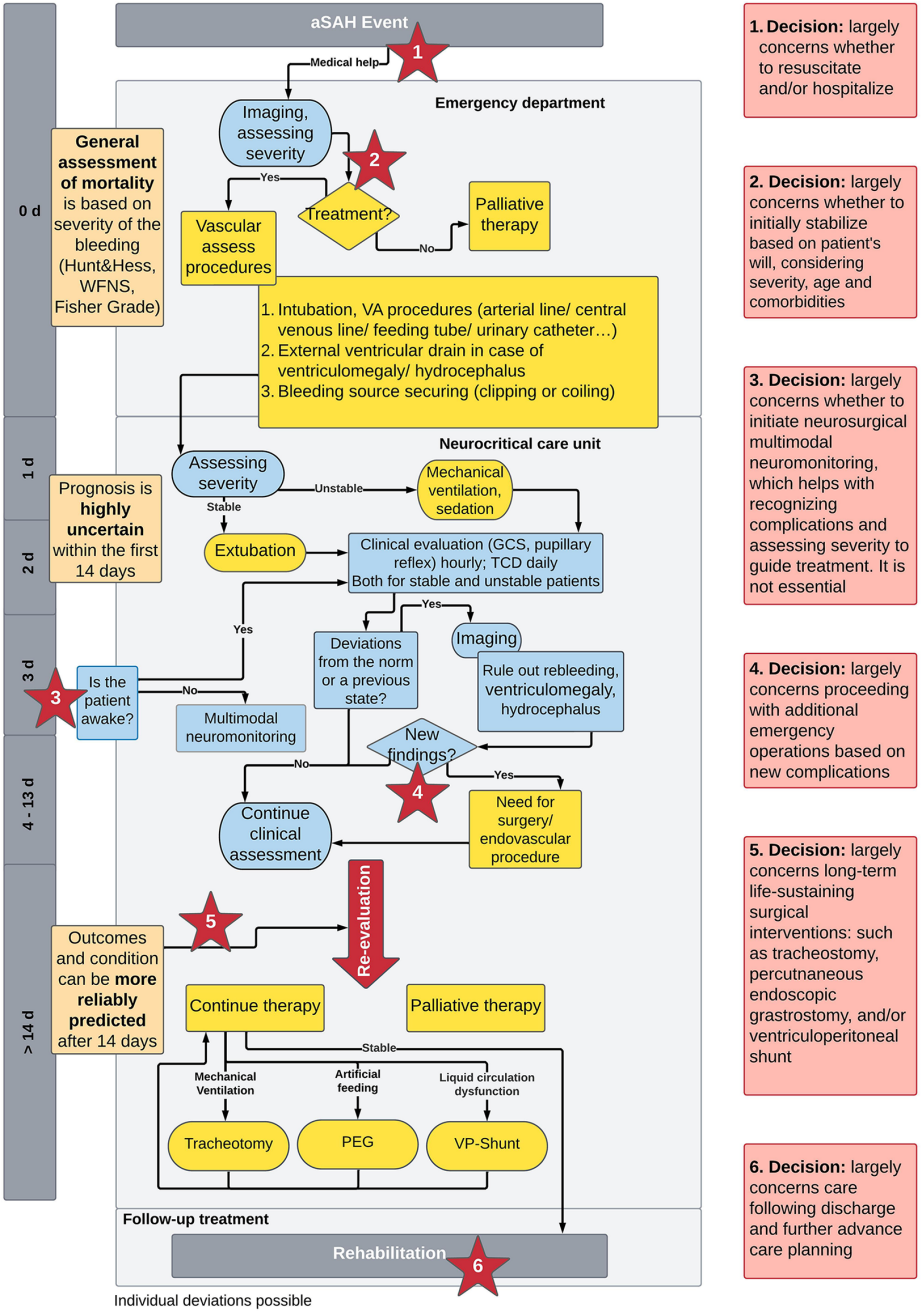
Timeline covering the two weeks period from initial bleed to stabilization for aSAH patients. Prognostic ability (orange), diagnostic efforts (blue), interventions (yellow), and preference- sensitive decision moments (red). d, day; GCS, glasgow coma scale; PEG, percutaneous endoscopic gastronomy; TCD, transcranial doppler; VP-shunt, ventriculoperitoneal shunt; WFNS, world federation of neurosurgical societies.

Drawing on existing guidelines for treating aSAH (12, 43), a recent qualitative study exploring patients’ experiences with aSAH (24), and additional input from clinicians in the neuro-critical care unit of the University Hospital Zurich, a timeline that highlights the critical moments for decision-making within the first two weeks following the initial bleeding event was developed. Figure 1 displays the timeline. The descriptions of the key decision-making moments depend on the patients' goals and values.

(1) Not all patients wish to be hospitalized following an emergency event. As such, the first preference-sensitive decision occurs immediately following the initial bleed and concerns the question of whether to initiate emergency aid and whether to transport the person experiencing aSAH to the hospital. Often, bystanders call for emergency help and medics proceed with stabilization and emergency transportation, but some people declare, or might have declared had they known about the option, not to be resuscitated and/or not to be hospitalized. Ideally, the person’s underlying motives for declining such interventions are known to clarify appropriate alternatives.
(2) Once the patient presents to the emergency department and aSAH has been diagnosed, it must be decided whether to secure the bleeding source to address the underlying conditions or whether to proceed with palliative treatment aimed at maximizing comfort and quality of life. This decision depends in large part on clinician judgement regarding what is appropriate and possible according to the severity of the bleeding and other factors affecting the person’s condition, such as age and comorbidities. Efforts at basic stabilization are often systematically initiated upon presentation to the emergency department and are extremely time sensitive (53). Nevertheless, it is important to identify as best as possible what burden of treatment the person is willing to undergo and what degree of cognitive and physical disability following they might be ready to accept.
(3) Around the third day of the patient’s stay in the neurocritical care unit if the patient has not awoken, the surgical option of multimodal neuromonitoring to guide treatment and better-detect vasospasm to prevent delayed cerebral ischemia is considered (54). Since such monitoring is not essential for treatment and requires an invasive procedure, surrogates are asked to decide whether to give consent. Patients requiring this kind of care also often require maximal intensive care and deep sedation with an associated higher risk of side effects. Given the intensive and burdensome nature of this care and the fact that loved ones have had more time to process the situation, this can be a key moment to revisit the question of whether to pursue further interventions and under what conditions it might align with the patient’s preferences to instead opt for more limited or palliative care.
(4) An inflection point occurs if there are new medical events or additional extracerebral or intracerebral complications needing emergency neurosurgical or neuroradiological interventions. In these instances, the prognosis may worsen and other interventions, some with higher levels of burden, may become relevant. While clinicians may decide that further intervention is not appropriate given the severity, often, deciding whether to proceed depends again on the degree of treatment burden and treatment outcomes the person would be willing to accept. Surrogates can be prepared in advance to consider such scenarios.
(5) Around the 14th day after the aSAH event when the risk for vasospasm and delayed cerebral ischemia is lower and prognosis more reliable, it may be necessary to consider long-term life-sustaining interventions. These interventions are planned operations, such as mechanical ventilation, artificial feeding, or the continuous draining of cerebrospinal fluid by a ventriculoperitoneal shunt. At this point a clearer- but still uncertain- prognosis can be presented of physical disabilities and – less accurately – of the cognitive deficits. This becomes an important moment to consider whether long-term life-sustaining interventions should be established or whether palliative care better fits with the understood quality of life the person has expressed as being worth living.
(6) Once patients are well-stabilized, choices are made regarding discharge from the ICU and attention can be given to anticipating preferred future care. Considerations regarding discharge include decisions about which forms and settings for rehabilitation are most appropriate, how families might structure support, and whether options such as nursing homes should be considered. Moreover, this is a critical moment to engage the patient and/or their loved ones in advance care planning to consider preferred care in the case of future health events, such as another rupture or a new aneurysm (12). Rehabilitation is a time to discuss the value of advance directives and the types of care questions that might arise in the future. First degree-family members can be made aware of their elevated risk for similar such conditions and counseled about how they might choose to engage with this knowledge, such as options for screening and treatment (55).

### Part three: how such timelines can support SDM in intensive care

There are many possible applications for integrating timelines that highlight preference-sensitive decision moments into digital tools to support SDM in intensive care. Their use can be preparatory- before an event; facilitatory - during an event; and reflective - following an event. These applications are described below:

#### Preparatory

Such timelines might be used to create more refined and enhanced advance directives. Specifically in cases where someone is high-risk for a condition and creating a focused advance directive, incorporating such timelines in advance directives might significantly enhance the preparedness of the person considering their preferences and the clinical utility of the resulting advance directive by clearly illustrating the flow of decisions and eliciting input regarding preference-sensitive decision moments with different options that affect outcome. Moreover, these timelines can be digitally embedded in such a way that they are interactive and capable of providing the user with more information about the contained terminology or procedures.

#### Facilitatory

Timelines can be embedded into electronic health records in a way that prompts clinicians with a reminder to engage the patient and their loved ones in SDM at certain stages of treatment. These reminders may be valuable to ensure that interventions aimed at treating, curing, and/or sustaining life are not maintained simply because they have been initiated. These prompts may support clinicians who are clear on the value of SDM but less sure when to initiate or revisit discussions in the flow of high-pressured decision-making.

Timelines can also be digitally shared with surrogate decision-makers once a patient has presented to the ICU as a way to prepare them for the moments when their input may be needed and support them in their role. As in the case of advance directives, these materials could be interactive with an informing function to better define terminology or explain procedures. Such timelines can complement discussions with clinicians, capturing information that has been discussed and illuminating questions that may need further clarification.

#### Reflective

Timelines may be important aids for evaluating care provided, both to determine if the choices aligned with a patient's preferences and to support clinicians in processing the experience. Such timelines can be used for formal or informal inquiry into retrospective agreement with received neurocritical care, elicited either from the patient him or herself and/or from those who played the role of surrogate depending on the person's capacity. Follow up questionnaires or interviews can be structured around the preference-sensitive decision moments. Moreover, debriefing difficult work situations is recommended for mitigating the risk of posttraumatic stress and burnout for ICU workers (56). Timelines may offer a framework to guide reflections on the care of critically ill patients in a way that supports clinicians in processing their role and responsibility.

## Discussion and conclusion

It is important to consider the potential challenges and risks of using timelines as well as their broader possible application. These timelines present granularity and complexity (57, 58). Preparing patients or surrogates to comment on specific interventions may lead to declarations that are ill-fitting, conflict with best clinical judgment, or do not truly reflect the patient's goals (59) due to limited understanding (60). Such timelines may be hard to understand without expertise and/or lead to feelings of overwhelm (61, 62). They may introduce fears concerning possible future events (59) or increase retrospective dissatisfaction. The appropriate use of timelines requires thoughtfulness about how they are presented, when, and to whom (59). Their design should take the audience into account (63, 64) and include input from users regarding comprehensibility, usability, and utility (65). Ideally, these timelines should aim to support collaboration (1, 4). Other professional groups may also benefit from their use, such as spiritual counselors, social workers and members of ethics committees (59).

There is broad applicability for these timelines outside the use case of aSAH (59). Following the high-level methodology outlined in Box 1, we suggest these timelines be developed for other conditions where patients may be unable to participate in decision-making, the patient's status is likely to evolve, and quick decisions must be made. These timelines may have an important role to play in multi-component advance decision aids, potentially supported by artificial intelligence (AI) in the future (66). There is a need for continued interprofessional collaboration amongst ethicists, clinicians, developers, designers, and intended audience to create effective tools that support SDM (65).

Box 1 **Methodology for developing timelines supporting SDM in intensive care.**

1. Screen existing clinical guidelines to determine standard care pathways for the designated illness or injury.
2. Draft an outline of the main treatment options and frequent complications, specifying when they tend to occur.
3. Gain input from health professionals, patients, and surrogates about moments when treatment decisions must be made that rely on patients' preferences. This can draw on questionnaires, interviews, evaluation of medical records, advance directives etc.
4. Mark preference-sensitive decision moments and describe the essence of the choice.

## Data Availability

The original contributions presented in the study are included in the article/Supplementary Material, further inquiries can be directed to the corresponding author.
